# Breathing out dental fear: A feasibility crossover study on the effectiveness of diaphragmatic breathing in children sitting on the dentist's chair

**DOI:** 10.1111/ipd.12958

**Published:** 2022-04-22

**Authors:** Martina Levi, Maurizio Bossù, Valeria Luzzi, Federica Semprini, Andrea Salaris, Cristina Ottaviani, Cristiano Violani, Antonella Polimeni

**Affiliations:** ^1^ 9311 Department of Psychology Sapienza University of Rome Rome Italy; ^2^ 9311 Department of Oral and Maxillofacial Science Sapienza University of Rome Rome Italy; ^3^ Neuroimaging Laboratory IRCCS Santa Lucia Foundation Rome Italy

**Keywords:** children, deep breathing, dental fear, diaphragmatic breathing, heart rate variability

## Abstract

**Background:**

Anxiety related to the dental context is a clinically significant challenge. In children, dental fear is often accompanied by disruptive and uncooperative behaviours that can render treatment difficult. Although techniques to reduce children's anxiety exist, many have not been formally evaluated.

**Design:**

Diaphragmatic breathing has been shown to reduce fear and anxiety, but few investigations have evaluated whether it can reduce dental anxiety in children. This crossover study tested the effectiveness and feasibility of diaphragmatic breathing in twenty children undergoing dental care.

**Results:**

Compared with the treatment as usual, such a simple technique had significant benefits on mood, self‐reported pain and autonomic balance, thus reducing sympathetic activation.

**Conclusion:**

Diaphragmatic breathing is a low‐cost, easy–to‐implement technique suitable for daily dental practice, and is a promising tool for reducing negative effect and physiological distress in children with dental anxiety that results in more cooperative behaviours and reduced visit time.

**Why this paper is important to paediatric dentists**

- Compared with the treatment as usual, diaphragmatic breathing during dental treatment had benefits on moods, self‐reported pain and physiological activation in children with dental anxiety.
- Diaphragmatic breathing reduced dental visit time in children with dental anxiety, likely due to an increase in cooperative behaviours.
- Diaphragmatic breathing represents a promising tool for reducing psychological and physiological distress in children with dental anxiety.

## 1 INTRODUCTION

Dental fear and dental anxiety are often used interchangeably in the literature and frequently appear under the umbrella term ‘dental fear and anxiety’.^1^ In both the Diagnostic and Statistical Manual of Mental Disorders^2^ and the International Statistical Classification of Diseases and Related Health Problems, 10th Revision,^3^ dental phobia is defined as persistent anxiety in relation to either clearly distinguishable situations/objects (such as drilling and injections) or dental situations in general.^1^ There are, however, conceptual differences between fear and anxiety. Dental fear is an emotional reaction to one or more specific threatening dental stimuli, whereas dental anxiety is a state of apprehension that something bad will happen in relation to dental treatment, together with a sense of losing control.

An individual's experiences of dental fear and dental anxiety can present along a continuum from very mild to extreme, but only a small proporotion of fearful individuals exhibit clinical dental phobia. Studies indicate that approximately 10% to 20% of the adult population report high dental anxiety with the majority developing it during childhood.^4^, ^5^ In Australia, dental fear affects approximately one in six adults.^6^ A similar prevalence is found in many Western countries around the world, such as Britain,^7^ France,^8^ Canada,^9^ Germany^10^ and Finland.^11^ Overall, dental anxiety has been ranked fifth among the most common causes of anxiety.^12^

### 1.1 Impact and consequences of dental fear

The impact of dental fear can be considerable. First, both fear and anxiety toward the dentist and dental treatment are significant contributors to the avoidance of dental care.^13^ Second, children and adults with high levels of dental fear are difficult to manage and require more treatment time and resources, and can lead to a stressful and unpleasant experience for both the patient and the dentist.^14^ Lastly, because of their avoidant behaviours, dentally anxious people frequently have poorer dental health.^15^, ^16^ Several studies provide a clear demonstration of the existence of oral health disparities in individuals with extreme dental fear compared with the general population.^17^, ^18^

In 1984, Berggren^19^ proposed that the relationship between dental anxiety and avoidance can lead to a vicious circle. Dental fear leads to the avoidance of dental care, which, in turn, results in a worsening of dental problems, requiring more intensive and potentially painful treatment, which then either reinforces or exacerbates fear and prolongs avoidance,^20^ subsequently causing poorer oral health. This process is also made worse by feelings of embarrassment and shame.^21^ Moreover, high dental anxiety has been shown to affect quality of life.^7^, ^22^

To reduce the development of avoidant and uncooperative behaviours, which can persist into adulthood, it is important to treat dental anxiety and fear in childhood.

Evidence shows how children in different cultures have similar concerns about dental procedures, with the most feared items of the Children's Fear Survey Schedule—Dental Subscale (CFSS‐DS^23^) being ‘dentist drilling’, ‘choking’ and ‘injection’.^24^ Nevertheless, the development of dental fear and anxiety is complex and there are clearly different exogenous and endogenous aetiological factors: negative past experiences, the media, role models, genetics, personality and intelligence, which have all been demonstrated to play a role.^25^, ^26^, ^27^, ^28^

Young children are particularly vulnerable to suffer from some degree of dental fear, particularly during the first visit; this fear typically decreases by visiting the dentist more often. In a small subgroup of children, however, such fear seems to persist into adulthood and becomes chronic.^29^

### 1.2 Identifying dentally anxious children

Three main methods are available to evaluate dental anxiety in children: (1) direct observation of the child's behavioural response or physiological state in the dental context; (2) parent‐report questionnaires; and (3) self‐report scales completed by the child.^30^ For a better understanding of the emotional state of children in the dental environment, however, additional objective assessments are required. Observational studies show that uncooperative behaviours do not always reflect the psychological state of children; at the same time, cooperative behaviors do not mean the child is not undergoing stress.

Anxiety and fear responses are usually accompanied by changes in the autonomic nervous system (ANS) activity; in fact, dental fear elicits increases in heart rate (HR) and sweating.^31^ An important measure of the interplay between sympathetic and parasympathetic nervous system functioning is the HR variability (HRV), defined as the variation in time intervals between consecutive heartbeats.^32^ In particular, higher resting HRV appears to be a marker of healthy physiological functioning, whereas lower resting HRV is associated with depression, anxiety and chronic stress and predicts disease risk and mortality.^33^ Hence, increasing HRV is beneficial for health and mood, and it has been shown to reduce symptoms of stress, anxiety, fear and depression.^34^, ^35^, ^36^, ^37^ In this regard, HR and HRV measurements could be valuable measures to assess children's fear in relation to dental treatment when no expressed signs of unease are present.

### 1.3 Non‐pharmacological management of children's dental fear

The successful management of dental anxiety in children may involve considerable time, effort and expertise; as reviewed by Anthonappa and colleagues,^38^ there are a wide range of strategies available to assist the dental team, such as the ‘tell–show–do technique’,^39^ music therapy,^40^ videos,^41^, ^42^ magic tricks,^43^ positive reinforcement,^44^ systematic desensitization^45^ and modelling.^46^

When behavioural management techniques are unsuccessful, however, children with high dental anxiety are often treated with pharmacological interventions, such as conscious sedation or general anaesthesia. Such pharmacological approaches facilitate the dental treatment to be performed, without addressing the etiology of dental anxiety, which can subsequently persist into adulthood.^47^ For this reason, psychological interventions applied to dental care are receiving increasing attention, though most studies have focused on adults.^18^, ^48^ In children, psychological strategies can enhance trust, increase feelings of control and help to develop adequate coping skills.^49^ To date, cognitive strategies appear to be effective, demonstrating a significant reduction in dental anxiety and an improvement in the quality of life of children attending a hospital dental service.^50^ Furthermore, 91% of patients report the reduction in their dental anxiety to be maintained one year later.^51^

It is important to consider whether those interventions can be feasibly integrated into dental practice, in the light of time and resource constraints of the dental team. Other barriers to the effective delivery of psychological services to children with dental fear are the associated costs and long waiting lists. Moreover, dentists themselves can be skeptical or unwilling to employ psychological techniques in their practice; thus, there is the need to provide specialists in paediatric dentistry with more information on effective practices to manage children's dental anxiety.^52^

### 1.4 Diaphragmatic breathing

An exercise that is believed to be of benefit to almost every fearful patient is relaxation through paced deep breathing.^53^ Diaphragmatic breathing has been used across a wide range of situations to reduce anxiety and perceived pain.^54^, ^55^ In the dental literature, the association between high levels of anxiety and increased pain perception is well established^56^: anxiety upregulates the sympathetic nervous system, which, in turn, decreases pain threshold.^57^ For example, Milgrom and colleagues in 2009^58^ conducted a study in which dentally anxious patients were taught to take slow and deep breaths, holding each breath for approximately 5 s, before slowly exhaling. This procedure was effective in reducing patients’ HR and making them feel noticeably more relaxed. Breathing techniques can be taught quite easily in the dental clinic and have demonstrated effectiveness in minimizing dental injection anxiety in adults.^59^

Diaphragmatic breathing reduces respiration frequency and maximizes the amount of blood gases through contraction of the diaphragm, expansion of the belly and deepening inhalation and exhalation, reducing stress and anxious states.^60^, ^61^ Evidence indicates that even a single breathing practice significantly reduces blood pressure and increases HRV^32^, ^62^ and oxygenation.^63^, ^64^

In the light of the close association between respiration, ANS activity and emotions,^65^ affective states can be changed through breathing by voluntary control.^66^ For this reason, diaphragmatic breathing is currently applied in clinical treatments for mental conditions, such as post‐traumatic stress disorder,^67^ phobias^68^ and other stress‐related disorders.

Diaphragmatic breathing is low cost and easy to teach to most children; it can be taught through verbal instructions, role modelling and imitation.^69^ For this reason, biofeedback‐assisted breathing training is used in the treatment of headaches, sleep disorders, recurrent abdominal pain, attention‐deficit disorder, epilepsy and anxiety in children.^70^ In a survey of children who participated in a biofeedback programme, 80% identified breathing practice as the component of the training they used the most and retained for the longest.^71^ Slow deep breathing is also described as an important intervention tool for distressed or angry children, for children who are anxious about athletic or test performance, or for those who feel generally tense.^72^

Despite the acknowledgement of its potential, there is a lack of studies implementing diaphragmatic breathing as a method to reduce dental anxiety in paediatric dentistry.

### 1.5 Objectives of this study

This crossover study tested the feasibility of a low‐cost and easy‐to‐teach procedure to reduce the pain and negative feelings of children undergoing dental treatment. We hypothesized reduced physiological and self‐reported measures of anxiety and pain in children undergoing diaphragmatic breathing versus treatment as usual (TAU). We expected the duration of the dental treatment to be shorter in the experimental condition than in the control condition, with the hypothesis that reduced anxiety would lead to more cooperative behaviours.

## 2 MATERIALS AND METHODS

### 2.1 Methods

The study was conducted at the Paediatric Dentistry Unit of the Department of Oral and Maxillofacial Science of Sapienza University of Rome, Italy.

#### 2.1.1 Participants

Twenty‐five children (14 males) undergoing two comparable dental procedures on different days were recruited. Due to the COVID‐19 pandemic, 5 children could not return for the second appointment; therefore, the final sample was composed of 20 children (11 males; mean age 9.2 [1.76] years) in a within‐subject design. Children with dental phobia, assessed via selected items of the Children's Fear Survey Schedule—Dental Subscale (CFSS‐DS^23^; eg, ‘*How afraid are you of the sight of the dentist's drill?’*), were not enrolled in the study (ie, exclusionary criterion). This choice was motivated by the fact that, just as blood phobia, dental phobia is characterized by a vasovagal response. In such cases, a further increase in parasympathetic function would not be recommended and be associated with the risk of triggering fainting.

The study was approved by the Institutional Review Board (IRB) of the Department of Psychology, Sapienza University of Rome, Italy (Prot. no. 0000940).

#### 2.1.2 Procedure

Children who met the inclusion criteria (age range between 7 and 13 years; dental procedures such as extraction or treatment of caries) and had an appointment at the Paediatric Dentistry Unit of the Department of Oral and Maxillofacial Science were invited to participate in the study. In the waiting room, caregivers who were accompanying the child were informed about the purposes and procedures of the study and, if interested, signed the informed consent.

Then, the child was given the CFSS‐DS to exclude the presence of dental phobia. If eligible, the child was assigned to the TAU or diaphragmatic breathing condition using computer‐generated random numbers (simple randomization). The child was subsequently invited to complete the other questionnaires, either in the waiting room or sitting on the dental chair, depending on dentist's time constraints. When the child was laying down on the chair, the three ECG electrodes were attached, and the respiration belt was put around the child's abdomen; then, 3 min of baseline physiological assessment was performed, and the baseline visual analogue scales (VAS) were administered. The equipment was well tolerated by children. The baseline resting period ensured that children could acclimate to the physiological recording equipment before the introduction of the intervention.

Children assigned to the experimental condition were then given the instructions for diaphragmatic breathing, and only when they were able to perform it, the dental treatment could start. Children assigned to the TAU condition were ready to normally start their dental care immediately after physiological baseline recording and VAS completion. The duration of dental treatment was recorded. When the dental procedure was over, children were asked to remain on the chair for a 3‐minute recovery assessment, while the VAS were delivered again. After the removal of the electrodes, children were then thanked for the participation and taken back to their caregivers in the waiting room. After several months, children came back for another appointment and underwent the experimental condition they were not assigned to during the first session. The second session was identical to the first, except for dispositional questionnaire, which were filled out only during the first appointment and the activity the child performed during the dental procedure (ie, diaphragmatic breathing or TAU).

The intervention was delivered by a graduate student in psychology (either ML or AS) under the supervision of a senior clinical psychophysiologist with expertise in breathing techniques (CO). To exclude any possible difference between the two dental visits, all the children saw the same dental practitioner (FS) for both appointments.

### 2.2 Materials and measures

#### 2.2.1 Questionnaires

During the first visit, children were asked to fill out the following self‐report dispositional questionnaires.

The CFSS‐DS^23^ comes from an 80‐item questionnaire designed to assess a variety of children's fears.^73^ The dental subscale is made up of 15 items requiring children to rate how frightened they are in different dental‐related situations or treatments (ie, ‘*dentist’* and ‘*injections’*). It is based on a Likert‐scale response format, ranging from 1 (not scared at all) to 5 (very scared). In this study, the CFSS‐DS has been used to exclude the presence of a proper dental phobia.

The Multidimensional Anxiety Scale for Children (MASC^74^) is a 39‐item 4‐point Likert self‐report scale to assess the presence of symptoms related to anxiety disorders in children and youth aged 8 to 19. The MASC has been cross‐validated in clinical and population samples.^75^ The main 4 factors (subscales) are as follows: (a) physical symptoms (ie, ‘*I have trouble breathing’*); (b) social anxiety (ie, ‘*I am afraid the other children will laugh at me’*); (c) separation anxiety (ie, ‘*I sleep next to someone of my family’*); and (d) harm avoidance (ie, ‘*I do my utmost to obey my parents and teachers’*). Answers range from 0 (never) to 3 (always).

The Children's Depression Scale (CDS^76^) is designed to assess the severity of depression covering an age range from middle childhood to late adolescence. The measure includes 66 items that comprise two scales: depression (48 items; eg, ‘*I often think I have done something wrong’*) and positive affective experience (18 items; eg, ‘*I feel happy’*). Responses range from 0 (very wrong; unlike me) to 3 (very right; like me).^77^

The Children's Response Style Questionnaire (CRSQ^78^) consists of 25 items, each of which describes a particular response to symptoms of depression. The items are grouped into three subscales: (a) rumination, including 13 items describing responses to depressed mood that are self‐focused (eg, ‘*Think about how alone you feel’*); (b) distraction, including 7 items describing responses to depressed mood that divert the individual's attention from sad mood (eg, ‘*Watch TV or play video games so you don't think about how sad you are’*); and (c) problem‐solving, including 5 items that describe strategies to overcome depressed mood (eg, ‘*Ask a friend/parent/teacher to help you solve your problem’*). Children are asked to indicate how often they respond in that particular way when they are feeling sad (0 = almost never, 1 = sometimes, 2 = often or 3 = almost always). Higher scores on each subscale indicate a greater tendency to engage in that particular response style.^79^

### 2.3 Visual analogue scales

Visual analogue scales were used to assess the current levels of happiness, fear, anger, sadness and pain from 0 (not at all) to 10 (very much). In this study, the VAS have been adapted to children; in fact, they consisted in coloured cartoons with smiles showing different levels of each emotional state. The VAS were administered before the dental treatment, namely during baseline physiological assessment and at the end of dental care procedures (recovery period).

### 2.4 Physiological assessment

A validated portable device (ProComp5 Infiniti; Thought Technology Ltd) was used to assess respiration amplitude and electrocardiography (ECG) to derive HR and HRV in the frequency domain: high frequency (HF‐HRV: 0.15–0.4 Hz) and the ratio between low frequency and HF (LF/HF).^80^ HF‐HRV reflects parasympathetic activity, whereas LF/HF reflects the dominance of sympathetic over parasympathetic activation.^80^

### 2.5 Interventions

#### 2.5.1 Diaphragmatic breathing

Children were first taught that they can control their breathing voluntarily. The word ‘belly breathing’ was used to help them to focus attention on their belly as they breathed. Children were encouraged to put one hand over the chest and the other hand over the abdomen and then breath normally, paying attention to the related changes in these body parts. Children were advised to imagine having in their belly a balloon of their favourite colour that inflated when they inhaled and deflated as they exhaled, or they were told to imagine blowing their air down through their legs to their feet. The correct breathing pattern was modelled with the experimenter inhaling and exhaling in phase with the child to make him or her exhale longer and shift to a diaphragmatic pattern.

#### 2.5.2 Treatment as usual

In the control condition, TAU was carried out. In this condition, children had just to lie down on the dental chair, while their physiological parameters were recorded, with no further instruction or any reference to breathing.

### 2.6 Data analysis

Given the lack of previous studies using this specific technique to reduce dental fear or anxiety in children, the sample size was computed based on the few studies conducted in this population using other behavioural approaches (eg, *n* = 22 per group^81^; *n* = 16^82^).

First, associations between socio‐demographic (age, sex) and dispositional (scores on the questionnaires) characteristics and the main outcome variables of the study at baseline were examined. For these analyses, the average of the two baseline periods was used.

To check whether participants effectively performed diaphragmatic breathing during the dental visit, a within‐subject general linear model (GLM) was performed on respiratory rate.

Then, a series of GLMs having Time (pre, during and post) and Condition (experimental and control) as within‐subjects variables, and the order of interventions as a covariate were performed on each physiological variable (HR, HF‐HRV and LF/HF‐HRV).

Subsequently, a series of GLMs having Time (pre and post) and Condition (experimental and control) as within‐subjects variables, and the order of interventions as a covariate were performed on each self‐reported variable measured through the VAS (fear, sadness, pain, anger and happiness).

Lastly, a GLM having Condition (experimental and control) as within‐subjects variable and the order of interventions as a covariate was performed on the duration of the dental treatment.

For all the GLM analyses, besides the significance, the effect sizes are reported through the indices Cohen's *d* or partial eta‐squared (*η_p_^2^*).

## 3 RESULTS

Table 1 illustrates means, standard deviations and ranges of scores on the dispositional questionnaires for the examined sample. As to baseline momentary affect, an inverse correlation emerged between age and self‐reported levels of sadness (*r* = −.55; *p* = .011). Scores on the social anxiety subscale of the MASC correlated with baseline levels of fear (*r* = .78; *p* = .002), with socially anxious children reporting to be more fearful at the beginning of the dental visit. Scores on the harm avoidance subscale correlated with baseline levels of anger (*r* = .48; *p* = .033) and sadness (*r* = .52; *p* = .018), with children prone to avoid risks and dangers reporting more anger and sadness at the beginning of the dental visit. Self‐reported fear at baseline also correlated with the rumination subscale of the CRSQ (*r* = .52; *p* = .019), with children characterized by a higher tendency to engage in ruminative thoughts also reporting higher levels of fear at the beginning of the dental visit. HF‐HRV at baseline negatively correlated with the social anxiety subscale scores of the MASC (*r* = −.50; *p* = .029) and with the rumination subscale of the CRSQ (*r* = −.51; *p* = .026), suggesting increased physiological activation in socially anxious children and ruminators. No other significant correlations emerged.

**TABLE 1:** Dispositional characteristics of the sample

	Mean (SD)	Range
CFSS‐DS	27.35 (7.52)	17–44
MASC physical symptoms	14.85 (5.75)	5–25
MASC social anxiety	11.05 (5.12)	2–20
MASC harm avoidance	11.3 (5.42)	1–21
MASC separation anxiety	18.8 (4.02)	12–26
CDS	11.85 (3.64)	6–18
CRSQ rumination	16.45 (10.9)	4–38
CRSQ distraction	9 (3.09)	5–17
CRSQ problem‐solving	8.05 (3.32)	2–14

Abbreviations: CDS, Children's Depression Scale; CFSS‐DS, Children's Fear Survey Schedule—Dental Subscale; CRSQ, Children's Response Style Questionnaire; MASC, Multidimensional Anxiety Scale for Children.

Table 2 illustrates means and standard deviations of the physiological and self‐reported measures before, during and after the dental procedure in the two examined conditions. The manipulation check confirmed a significant Time × Condition interaction for respiration rate (*F*_2,36_ = 21.57; *p* < .0001), with the experimental condition showing a decrease in respiration rate during the dental procedure (*t* = 4.69; *p* < .0001) and no changes in the control condition (*t* = 1.03; *p* = .316).

**TABLE 2:** Physiological and self‐report measures before, during and after the dental procedure in the experimental (diaphragmatic breathing) and control conditions (*n* = 20)

	Diaphragmatic breathing			Control condition			Time × Condition (*p* Value)
	Baseline	Dental procedure	Recovery	Baseline	Dental procedure	Recovery	
HR (bpm)	92.1 (12.8)	89.7 (12.6)	89.7 (13.7)	94.1 (14.8)	95.5 (14)	94.6 (13.9)	.058
HF‐HRV (ms^2^)	921.5 (1022.9)	1355.7 (1854.2)	1167.2 (1278)	975.5 (1064.3)	1006.4 (1486.6)	1618.4 (3173.9)	.33
LF/HF	1.1 (0.6)	0.8 (0.4)	1.1 (1.1)	1.1 (0.6)	1.4 (0.6)	1.2 (1.1)	.002
Respiration rate	13.9 (1.4)	12.8 (1.1)	13.9 (1.5)	13.9 (1.2)	15.3 (1.5)	14.2 (1.2)	<.0001
Fear (VAS)	4.2 (3.5)	/	4.1 (4.4)	3 (2.8)	/	4 (4.1)	.024
Sadness (VAS)	2.5 (2.2)	/	0.8 (2.4)	2.9 (2.8)	/	3 (2.9)	.023
Pain (VAS)	0.7 (1.6)	/	1 (2.5)	0.8 (1.7)	/	2.7 (2.9)	.007
Anger (VAS)	0.7 (1.3)	/	0.9 (1.9)	1.2 (2.1)	/	2.3 (2.7)	.025
Happiness (VAS)	8.2 (1.9)	/	8.9 (2.6)	7.2 (2.8)	/	6.6 (2.9)	.28
Time taken (min)	37.8 (21.6)			42.5 (22.7)			.43 (Condition)

Abbreviations: HF‐HRV, high‐frequency heart rate variability; HR, heart rate; LF/HF, ratio of low frequency to high frequency; VAS, visual analogue scales.

As to physiological measures, a marginally significant Time × Condition (*F*_2,36_ = 3.07; *p* = .058, *η_p_^2^* = 0.14) interaction emerged for HR, with a trend towards a HR decrease in the experimental condition (*d* = 0.19) and a HR increase in the control condition (*d* = 0.10). Thus, whereas children undergoing TAU became more physiologically activated during the dental treatment, the abdominal breathing exercise was effective in reducing HR.

A significant Time × Condition interaction emerged for LF/HF (*F*_2,36_ = 7.11; *p* = .002, *η_p_^2^* = 0.27), with a reduction from baseline to the dental procedure in the experimental condition only (*t* = 2.32; *p* = .032; *d* = 0.59), and no differences between recovery and the other two conditions (baseline and dental visit) in both conditions. In other words, the decreased sympathetic over parasympathetic dominance produced by abdominal breathing was maintained in the post‐dental treatment recovery period. No other main effects or interactions emerged.

As to self‐reported measures, significant Time × Condition interactions emerged for self‐reported levels of:

- Fear, (*F*_1,18_ = 6.09; *p* = .024, *η_p_^2^* = 0.25), with a marginally significant increase from pre‐ to post‐dental visit in the control condition only (*p* = .044; *d* = 0.28); an increase in self‐reported levels of fear appeared only when children were not performing abdominal breathing.
- Sadness, (*F*_1,18_ = 6.22; *p* = .023, *η_p_^2^* = 0.26), with a significant reduction from pre‐ to post‐dental visit in the experimental condition only (*p* = .041; *d* = 0.73). Children performing diaphragmatic breathing reported feeling less sad at the end of the visit, whereas this did not happen in the TAU condition.
- Pain, (*F*_1,18_ = 9.15; *p* = .007, *η_p_^2^* = 0.34), with a significant increase from pre‐ to post‐dental visit in the control condition only (*p* = .03; *d* = 0.80). This is noticeable, because it means that abdominal breathing was effective in dampening the pain surge usually associated with dental care.
- Anger, (*F*_1,18_ = 5.97; *p* = .025, *η_p_^2^* = 0.25), with a marginally significant increase from pre‐ to post‐dental visit in the control condition only (*p* = .09; *d* = 0.45): when they did not practise diaphragmatic breathing, children reported feeling angrier at the end of the dental visit.

No significant main effects or interactions emerged for self‐reported levels of happiness.

The GLM having the duration of the dental treatment as outcome yielded a main effect of Condition (*F*_1,18_ = 4.73; *p* = .043, *η_p_^2^* = 0.21), with reduced length of the visit in the experimental compared with the control condition (37.8 [21.6] vs. 42.5 [22.7], respectively; *p* < .0001; *d* = 0.21). Hence, diaphragmatic breathing made dental visits shorter.

## 4 DISCUSSION

Overall, the current results suggest that diaphragmatic breathing can be a promising intervention to (a) enhance physiological relaxation (as indexed by decreased HR and LF/HF), (b) decrease self‐reported levels of pain and (c) increase subjective well‐being in children undergoing dental treatment. Moreover, these beneficial effects were associated with a significant reduction in the duration of the dental intervention. A possible explanation could be that a decrease in levels of pain, as well as in subjective and physiological levels of fear, led to increased children's cooperative behaviour.

Importantly, the difference in respiration rate between the two conditions confirmed that children correctly followed the protocol for diaphragmatic breathing, pointing to the feasibility of implementing this technique in an ecological environment during dental treatment.

Anxiety is characterized by increased sympathetic and reduced parasympathetic modulation of the heart.^68^ Diaphragmatic breathing was effective in counterbalancing these effects, favouring a reduction in sympathetic over parasympathetic nervous system dominance. The present results are in line with a study conducted in adults by Milgrom and colleagues^58^ who taught dentally anxious individuals to take slow and deep breaths, reducing their HR and making them feel noticeably more relaxed. A recent meta‐analysis has shown that slow breathing—performed by HRV biofeedback—can be effectively used to reduce anxiety and stress^34^ with effects of large size (Hedges' *g* > 0.8). Effects of the same size have been reported for improvements in emotional and physical health and performance.^83^ Neuroimaging data support the view that slow breathing acts via vagal pathways by increasing the functional connectivity between prefrontal and limbic networks involved in the emotion regulation.^84^, ^85^

Well‐replicated evidence now exists, suggesting that increased parasympathetic control of the heart is associated with a reduction in perceived stress, depression, anxiety and fear.^34^, ^35^, ^36^, ^37^ It should be noted, however, that in this study, the lack of significant effects on HF‐HRV points to a decrease in sympathetic activation (lower LF/HF) rather than pointing to an increase in parasympathetic activation. Together with the reduction in sympathetic dominance caused by diaphragmatic breathing, participants reported to feel less pain, fear, sadness and (although only marginally significant) anger. Again, this is in accordance with the reported effectiveness of relaxation breathing in reducing anxiety and perceived pain in adults.^55^ In the dental context, Morarend and colleagues^59^ also supported the effectiveness of diaphragmatic breathing in decreasing dental anxiety and negative feelings towards a dental injection in adults. Despite the absence of studies conducted in children within the dental context, the use of breathing techniques in a developmental population appears to be helpful for the management of acute pain^86^, ^87^ or anxiety about sport or test performance.^72^

Notably, the application of a simple breathing technique was effective in reducing the duration of the dental treatment. This has important clinical implications, given that such a technique is easy to teach once, without the need to repeat the instructions during the subsequent visits. This implies that reducing perceived and physiological dental anxiety is also likely to increase compliance and ultimately dental health.^14^

Keeping in mind the limitation of a feasibility study and the need for replication, the current results provide insights for future investigations into the topic. For example, the relationship between the dispositional tendency to ruminate and self‐reported fear before the dental treatment could be interpreted as a possible factor implicated in the maintenance and worsening of dental anxiety. In other words, children's tendency to perseverate on their negative feelings could strengthen their fearful thoughts and the perception of dental care as threatening. Similarly, when children with higher dispositional levels of risk avoidance cannot escape from what they perceive as a harmful—as in the case of dental treatment—they might tend to feel angrier and sadder. Future studies should disentangle whether socially anxious children are more scared by the dental procedure itself or by the dental context in general (eg, being in the focus of the attention). Overall, the significant correlations that emerged in this study suggest that dispositional factors such as social anxiety, the tendency to engage in depressive rumination and harm avoidance could be useful for the early identification of vulnerable children in terms of dental anxiety, and therefore the population who would benefit the most from a diaphragmatic breathing training before a dentist appointment. A previous study on vulnerability factors focused on socio‐economic variables or being the only child or dispositional characteristics such as introversion.^88^ Thus, this study adds to the existing literature highlighting the need to extend the dispositional factors that are the object of investigation in the field.

Being the first on this topic, this study intended to be preliminary, and therefore, its main limitation concerns the examined sample size. The post hoc power analysis was performed with G Power 3.^89^ Based on the effect size found for subjective levels of fear (*η_p_* ^2^ = 0.25), the number of participants (*n* = 20) and the significance value (*p* < .05), the analysis revealed that the power of this study was 1‐*β* > 0.80 in an analysis of variance (ANOVA) with Time and Condition as within‐subject factors.

Another limitation involves the use of single items to assess complex multidimensional outcomes such as pain, because of time constraints. Importantly, the significant Time‐by‐Condition interaction found for respiration rate is important to exclude those children who first experienced the experimental condition and who have implemented the acquired breathing skills to feel better during their subsequent visits.

The major strength is ecological validity, as the study was conducted in a real workplace environment, and not in an experimental setting such as a laboratory. The ideal intervention should consider dentists' time constraints and ease of movements. Diaphragmatic breathing not only met these requirements but is also not associated with additional costs.

Proper randomized controlled clinical trials to test whether these promising results are replicated in a larger sample and more controlled conditions are aimed for. In future studies, it would be invaluable to disentangle the potential for carryover effects; given that a crossover design is ineffective at testing a carryover hypothesis, the use of mixed‐effect models to estimate carryover parameters would be highly recommended.^90^

Investigations into the application of promising treatments would require a multidisciplinary approach, involving both experts in psychological interventions and specialists in paediatric dentistry. Although such endeavours can be challenging, this effort is needed to improve our ability to deal with children's dental fear and to prevent the long‐term avoidance observed in adults with dental anxiety.

## CONFLICT OF INTEREST

The authors have no conflict of interests with respect to their authorship or the publication of this article.

## AUTHOR CONTRIBUTIONS

MB, VL, CO, CV and A.P conceived the ideas; ML, FS and AS collected and analysed the data; and ML and CO led the writing.
